# Management of Neglected Post-traumatic Bilateral Facet Dislocation of Sub-axial Cervical Spine: A Case Series

**DOI:** 10.31729/jnma.4912

**Published:** 2020-06

**Authors:** Rudra Prasad Marasini

**Affiliations:** 1 Department of Orthopedics and Trauma Surgery, National Trauma Center, National Academy of Medical Sciences, Kathmandu, Nepal

**Keywords:** *decompression*, *neurology*, *spinal injuries*

## Abstract

Neglected bilateral facet dislocation of the lower cervical spine is a rare condition and found mostly in developing countries like Nepal. Delayed presentation makes treatment more challenging concerning decompression, reduction, neurological recovery, and overall outcome. We managed three cases of bilateral facet dislocations of the fifth-sixth-seventh cervical vertebra level presented after three months of injury. All of those were treated surgically by combined anterior-posterior-anterior approaches with the same principle. One patient had a complete neurological recovery, the second one recovered partially with few long-term complications and the third one did not improve at all.

## INTRODUCTION

Delayed presentation of post-traumatic sub-axial cervical spine injury with bilateral facet dislocations are found mainly in the developing countries due to poor socioeconomic conditions, non-availability of nearby services, and lack of awareness.^1^ Management of these injuries is often challenging because of the difficult reduction and associated various intra-operative and post-operative complications.^2^ Several surgical approaches have been suggested, including anterior, posterior, and combined anterior-posterior-anterior approaches.^3^ The anterior-posterior-anterior approach for the decompression, reduction, stabilization, and fusion of these neglected traumatic dislocations is the method of choice in achieving the best sagittal alignment with less risk of iatrogenic neurological injury, better postoperative neurological recovery and overall long term outcome.^4^

## CASE REPORTS

**CASE 1**

**Figure 1:**
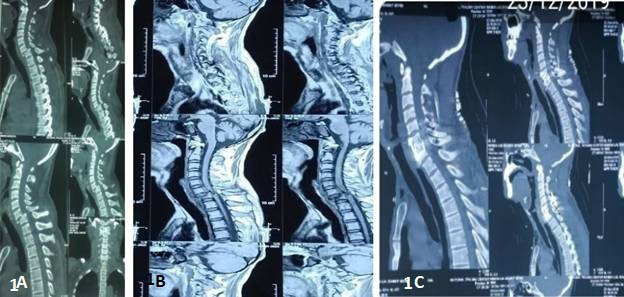
(A) Preoperative CT-Scan showing the fracture-dislocation of the C6-7 level. (B) Pre-operative MRI with significant anterior cord **compression. (C) Postoperative CT-Scan showing** adequate decompression, reduction, and fixation.

A 45-year-old male, from the Midwestern region of Nepal, presented in the emergency with the weakness of bilateral upper and lower limb for 4 months. He gave a history of fall from the tree (approximately 20 m height) and sustained injury over the neck and managed by the nearby hospital conservatively but he gradually developed weakness. On neurological examination, he had American Spinal Injury Association Impairment Scale (ASIA-C) neurology. Preoperative CT-Scan (Figure 1A) shows the fracture of 7^th^cervical vertebrae with bilateral facet dislocation at the C6-C7 level and Magnetic Resonance Imaging (MRI) shows significant cord compression (Figure 1B). He was diagnosed as a neglected fracture-dislocation of the C6-7 level with ASIA-C neurology. The patient admitted and kept on skull traction for one week but the reduction could not be achieved.

Anterior decompression by partial corpectomy of C7 vertebrae, posterior reduction by partial facetectomy, stabilization, and fusion by using lateral mass screws and bone graft, anterior cervical fusion by using a mesh cage, cancellous bone graft, and anterior cervical plating was done by combined Anterior-Posterior-Anterior (APA) approach. Immediately after surgery neurology was deteriorated to ASIA-B. Postoperative CT-Scan (Figure 1C) confirmed the adequate decompression and proper placement of hardwires. The patient was followed up to 6 months at a monthly interval and found that neurology was gradually recovered to normal by the end of three months.

**CASE 2**

**Figure 2:**
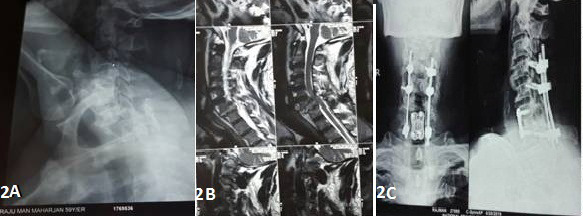
(A) Preoperative lateral x-ray of the cervical spine with fracture-dislocation of the C6-7 Level. (B) Preoperative MRI of the cervical spine with cord compression at the C6-7 level. (C) Postoperative AP and lateral x-rays of the cervical spine with anterior cervical plate, bone graft, lateral mass screws, and rod insitu.

A 59-year-old male, from the western region of Nepal, presented in the emergency with complain of pain and stiffness of neck and weakness of bilateral upper and lower limbs for 3 months. He had a history of falls from the tree and sustained neck injury 3 months back followed by the gradual development of weakness. On examination, he had ASIA-C neurology and a decreased neck range of motion. Pre-operative X-ray (Figure-2A) shows bilateral facet dislocation at the C6-C7 level and MRI (Figure 2B) shows significant cord compression at the same level. He was diagnosed as a neglected bilateral facet dislocation of the C6-7 level with ASIA-C neurology. The patient was admitted and kept on skull traction for 1 week and operated by the APA approach. Anterior cervical decompression, posterior reduction, stabilization and fusion, anterior cervical fusion by using tri-cortical iliac crest bone graft, and anterior cervical plating was done (Figure 2C). The patient developed acute renal failure postoperatively, which was managed by the nephrology team. Follow up was done at a monthly interval, by the end of 6 months, neurology was improved to ASIA-D with some myelopathic changes (Japanese Orthopedics Association Score = 15).

**CASE 3**

**Figure 3:**
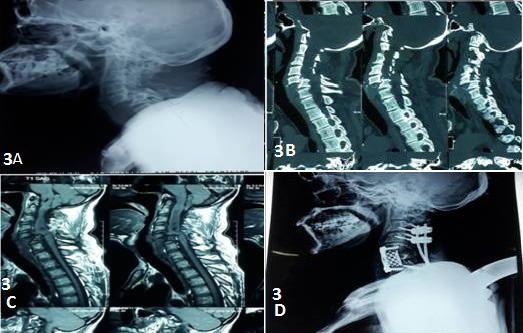
(A) Preoperative lateral X-ray of the cervical spine showing complete dislocation at the C5-6 level. (B) Preoperative CT scan of the cervical spine with complete bilateral facet dislocation C5-6 level. (C) Preoperative MRI showing significant cord compression at the C5-6 level. (D) Postoperative lateral x-ray showing anterior cervical plate and mesh cage with lateral mass screws and rod in situ.w

A 40 year male from eastern Nepal presented in the emergency with weakness of bilateral upper and lower limb for 3 months. He had a history of falls from tree 3 months back with sustained injury over the neck. On examination, he had a weakness of bilateral upper and lower limb with bowel and bladder involvement. Preoperative X-ray (Figure 3A), CT-Scan (Figure 3B), and MRI (Figure 3C) were done and diagnosed as neglected bilateral facet dislocation of C5-6 level with American Spinal Injury Association Impairment Scale (ASIA-A) neurology. He was kept on traction for two weeks and anterior cervical decompression, bilateral facetectomy, and laminectomy were done but the reduction couldn’t be achieved. Posterior stabilization and fusion by using lateral mass screws and cancellous bone graft were performed. Corpectomy of C6 vertebrae and in situ fixation and fusion by using mesh cage and bone graft and anterior cervical plating (Figure 3D) was performed by using combined APA. The patient was kept on follow up monthly and rehabilitated aggressively but no neurological improvement was found till the end of 6 mons of follow up.

## DISCUSSION

Bilateral facet dislocation of the sub-axial cervical spine is highly unstable injury, caused by flexion-distraction force, and most of the patients present with some neurological deficit.^5^ Cervical spine injuries, mainly at the cervicothoracic junction are frequently missed due to misinterpretation of radiographs, inadequate radiographs, and lack of suspicion. Our first case was also missed at the initial presentation. Close reduction, early decompression, and fixation are crucial for a better neurological outcome but in developing countries like Nepal, some patients could not undergo early surgery due to lack of infrastructure, awareness, and poor socioeconomic status.^6^ The reduction is always difficult in the neglected dislocations, we did the direct posterior reduction in the first two cases but this could not be achieved even after traction and surgery in the third case. Type of surgical approach (anterior, posterior, or combined) depends on the initial neurology, post-traction reduction status, presence of prolapsed intervertebral disc, and integrity of the disco-ligamentous complex.^5,6^ We followed a combined anterior-posterior-anterior approach to achieve adequate decompression, reduction, stabilization, and fusion. Initially, anterior surgery was performed to remove the fusion mass between vertebral bodies and to achieve complete discectomy.^1^

Without prior discectomy, not only makes the reduction more difficult but also increases an inherent risk of cord compression by a prolapsed disc during the reduction maneuver.^7^ Decompression is mandatory even in the late reported cases, where there is no evidence of cord transaction.^8^ Partial facetectomy and laminectomy have to be performed before reduction and stabilization in the second stage and anterior cervical fusion at the last. In the neglected case of long duration as in our third case, corpectomy of the lower vertebrae and in-situ fusion by using mesh cage and bone graft would be the only option remains when the reduction is not possible.^9,10^ We achieved complete neurological recovery in the first case, partial recovery in the second case, and no recovery at all in the third case. Overall outcome and the neurological recovery depends on the severity of the primary injury, the timing of surgery, adequacy of decompression, reduction and the stability.^10^

**Consent: JNMA** Case Report Consent Form was signed by the patient and the original article is attached to the patient’s chart.

## Conflict of Interest:

**None.**
